# Green Synthesis and Morphological Evolution for Bi_2_Te_3_ Nanosystems via a PVP-Assisted Hydrothermal Method

**DOI:** 10.3390/nano13212894

**Published:** 2023-11-01

**Authors:** Fang Zhou, Weichang Zhou, Yujing Zhao, Li Liu

**Affiliations:** 1 Department of Criminal Science and Technology, Department of Foundation Course, Hunan Police College, Changsha 410138, China; zhoufang@hhtc.edu.cn; 2 School of Physics and Electronics, Synergetic Innovation Center for Quantum Effects and Application, Key Laboratory of Low-Dimensional Quantum Structures and Quantum Control of Ministry of Education, Key Laboratory for Matter Microstructure and Function of Hunan Province and Institute of Interdisciplinary Studies, Hunan Normal University, Changsha 410081, China; 3 School of Physics, Electronictechnology and Intelligent Manufacturing, Huaihua University, Huaihua 418008, China; 4 School of Mathematics, Computer Science and Engineering, University of London, London EC1V 0HB, UK; liuliedsxps@gmail.com

**Keywords:** two-dimensional nanomaterial, Bi2Te3 hexagon nanosheet structure, PVP-assisted hydrothermal method

## Abstract

Bi${}_{2}$Te${}_{3}$ has been extensively used because of its excellent thermoelectric properties at room temperature. Here, 230–420 nm of Bi${}_{2}$Te${}_{3}$ hexagonal nanosheets has been successfully synthesized via a “green” method by using ethylene glycol solution and applying polyvinyl pyrrolidone (PVP) as a surfactant. In addition, factors influencing morphological evolution are discussed in detail in this study. Among these parameters, the reaction temperature, molar mass of NaOH, different surfactants, and reaction duration are considered as the most essential. The results show that the existence of PVP is vital to the formation of a plate-like morphology. The reaction temperature and alkaline surroundings played essential roles in the formation of Bi${}_{2}$Te${}_{3}$ single crystals. By spark plasma sintering, the Bi${}_{2}$Te${}_{3}$ hexagonal nanosheets were hot pressed into solid-state samples. We also studied the transport properties of solid-state samples. The electrical conductivity σ was 18.5 × 10${}^{3}$ Sm${}^{-1}$ to 28.69 × 10${}^{3}$ Sm${}^{-1}$, and the Seebeck coefficient *S* was −90.4 to −113.3 µVK${}^{-1}$ over a temperature range of 300–550 K. In conclusion, the observation above could serve as a catalyst for future exploration into photocatalysis, solar cells, nonlinear optics, thermoelectric generators, and ultraviolet selective photodetectors of Bi${}_{2}$Te${}_{3}$ nanosheet-based photodetectors.

## 1. Introduction

**Figure 1:**
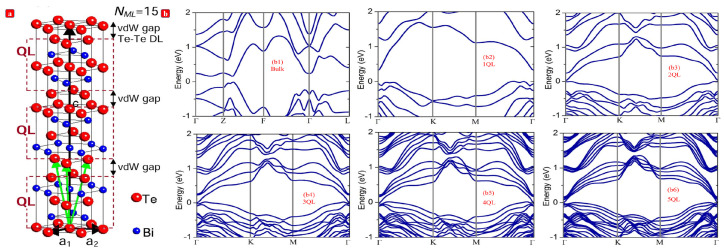
(**a**) Hexagonal unit cell of Bi${}_{2}$Te${}_{3}$ with the base vectors indicated by the black arrows. The unit cell consist of 15 atomic lattice planes that are grouped in three quintuple layers (QL) with Te${}_{\left(1\right)}$-Bi-Te${}_{\left(2\right)}$-Bi-Te${}_{\left(1\right)}$ stacking. The quintuple layers are van der Waals bonded to each other by a Te-Te double layer (van der Waals gap). The green arrows indicate an alternative definition of the crystal structure using rhombohedral base vectors. (**b**) Band structures of the bulk Bi${}_{2}$Te${}_{3}$ (b${}_{1}$) and Bi${}_{2}$Te${}_{3}$ nanofilms with five different thicknesses: 1QL (b${}_{2}$), 2QL (b${}_{3}$), 3QL (b${}_{4}$), 4QL (b${}_{5}$), 5QL (b${}_{6}$).

Bismuth telluride (Bi${}_{2}$Te${}_{3}$)-based alloy was the first thermoelectric (TE) material to be discovered, and it is the most maturely investigated and widely used TE material [1,2,3,4,5]. The rhombohedral crystal structure of Bi${}_{2}$Te${}_{3}$ belongs to the R$\bar{3}$m(166) space group symmetry [1]. There are five atoms per unit cell in the Bi${}_{2}$Te${}_{3}$. This compound contains a sandwich structure of the five layers [Te${}_{\left(1\right)}$-Bi-Te${}_{\left(2\right)}$-Bi-Te${}_{\left(1\right)}$], which are held together by van der Waals force in Figure 1a. Therefore, Bi${}_{2}$Te${}_{3}$ crystal grows faster in the a or b axis direction than in the c-axis direction, forming a hexagonal plates morphology at the end. Bi${}_{2}$Te${}_{3}$ nanoplates have an orientation along the (001) direction by the HRTEM image [6,7]. The ab initio electronic band structures of Bi${}_{2}$Te${}_{3}$ bulk material and nanofilms with various thicknesses are shown in Figure 1b. The spin-orbit interaction was taken into account in the calculation, which was proved to be essential for calculating the electronic structures of Bi${}_{2}$Te${}_{3}$, and our results for bulk Bi${}_{2}$Te${}_{3}$ (Figure 1(b${}_{1}$)) are in good agreement with the data reported in previous work. As shown in Figure 1(b${}_{2}$–b${}_{6}$), band structures of the Bi${}_{2}$Te${}_{3}$ nanofilms are similar to each other except for the significant difference in band gap and energy spacing between adjacent bands [8,9].

Among the state-of-the-art TE materials, Bi${}_{2}$Te${}_{3}$-based compounds are well known as the best materials for near room-temperature applications and the research on nanostructured Bi${}_{2}$Te${}_{3}$-based compounds is increasing [6,7,8,9]. As the most popular candidate for TE power generation and refrigeration [10], quintuple-layered Bi${}_{2}$Te${}_{3}$ is also known as a topological insulator (TI) [11], with an insulating bulk and metallic surface states protected by time-reversal symmetry [12], meaning charge carriers are not backscattered by nonmagnetic impurities and defects [13].

Recent research has shown that ZT of thermoelectric materials typically stays around 1.0 [14,15,16,17,18,19,20]. The conversion efficiency of Bi${}_{2}$Te${}_{3}$ is low, but low power applications are gradually gaining the attention of researchers [21,22,23,24,25,26,27,28]. Currently, a wide range of demands have been developed for low-power generation and cooling technologies, the applications of which primarily operate in room- or low-temperature environments, such as micro-motor power systems, IoT wireless sensing modules, and wearable intelligent samples [29,30,31,32,33,34,35,36,37].

Bi${}_{2}$Te${}_{3}$-based alloys have more promising candidates for application among numerous thermoelectric materials systems [38,39,40,41,42,43,44]. To create a high-efficiency TE material, the Seebeck coefficient and electrical conductivity should be enhanced while the thermal conductivity is minimized–in this way, the values for electrical conductivity and the Seebeck coefficient are obtained. Thus, studying the synthesis of Bi${}_{2}$Te${}_{3}$ and their solid solutions is necessary [45,46,47,48,49,50,51,52].

For the last several years, studies have focused on “green” chemistry for the design of chemicals and processes that reduce or eliminate the use or generation of hazardous substances [18,19,20,53,54,55]. Green synthesis is a chemical process that reduces or eliminates the use of hazardous chemicals and materials in the production of new chemicals and materials [56,57,58]. Green chemistry is also known as sustainable synthesis or eco-friendly synthesis [59,60]. In addition, green syntheses typically employ one or more of the following strategies: the use of renewable feedstocks, the use of environmentally benign reagents and solvents, the minimization of waste, and the maximization of atom economy. However, these approaches involve a strong reducing agent such as NaBH${}_{4}$, N${}_{2}$H${}_{4}$ and H${}_{2}$O${}_{2}$ [17,18,61,62]. Thus, developing environmentally friendly agents and nontoxic environments for chemical reaction is necessary, which is an important consideration in green synthesis.

To date, a hydrothermal method has been proven to be a feasible method to change the morphology and size of the synthesized Bi${}_{2}$Te${}_{3}$ nanostructures with low equipment and operating requirements, low cost, and low synthesis temperatures. Considerable effort has shown the synthesis of Bi${}_{2}$Te${}_{3}$, Bi${}_{2}$Se${}_{3}$, Sb${}_{2}$Te${}_{3}$, BiTe, and PbTe nanomaterials by using a solvothermal method [13,19,20,21,22,23,24,25,26,34,35,36,61,62,63,64]. Some studies reported the effect of the morphology of the products, but the synthesis method was not “green”, and some reported that the synthetic method was “green,” but the morphological changes were not reported. Other studies also reported that the synthesis method was “green” and tackled morphological changes, but the reaction efficiency was low [43,44,45,48,49,50,51]. However, a few papers on the impact of all response variables on morphology are found [19,20,21,26].

At present, we reported green, simple, and pure Bi${}_{2}$Te${}_{3}$ nanosheets by solvothermal treatment using ethylene glycol (EG), and influencing factors such as the reaction temperature, surfactants, and molar mass of NaOH were discussed in detail. The electrical conductivity σ ranged from 18.5 × 10${}^{3}$ Sm${}^{-1}$ to 28.69 × 10${}^{3}$ Sm${}^{-1}$, and the Seebeck coefficient *S* ranged from −90.4 to −113.3 µVK${}^{-1}$ over a temperature range of 300–550 K.

## 2. Experimental Sections

### 2.1. Preparation of Bi${}_{2}$Te${}_{3}$ Nanosheets

All the chemicals used for the synthesis of the Bi${}_{2}$Te${}_{3}$ nanosheets in this work are analytical grade without further purification. The normal synthesis procedure is described as follows: TeO${}_{2}$ (3.0 mmol, AR, Aladdin, Shanghai, China), BiCl${}_{3}$ (2.0 mmol, AR, Aladdin, Shanghai, China), NaOH (20.0 mmol, AR, Sino Pharm, Shanghai, China), and polyvinyl pyrrolidone (PVP, K-30, 1.0 g, AR, Sino Pharm, Shanghai, China) were dissolved in EG (100.00 mL, AR, Aladdin, Shanghai, China). The reaction kettle was heated to 180 °C and kept at that temperature for 36 h. After the reaction, the products were naturally cooled to room temperature. Next, the products were separated by centrifugation; then washed several times with deionized water, acetone, and anhydrous ethanol; dried at 60 °C for 6 h; and stored for various characterizations.

### 2.2. Sample Characterization

Using X-ray powder diffraction, the crystallographic phase structure of an as-prepared sample was determined (XRD, Bruker D8 Advance, Bruker, Mannheim, Germany) Using a scanning electron microscope, the size and shape of the finished product were determined (SEM, FEI NovaSEM-450, Baltimore, MD, USA) By using energy-dispersive X-ray (EDX) spectroscopy, the chemical composition and elemental mapping were studied. TEM (FEI Tecnai F20, Baltimore, MD, USA) operating at 200 kV was used to characterize the transmission electron microscopy (TEM) picture, the high-resolution transmission electron microscope (HRTEM) image, and the selected area electron diffraction (SAED) pattern. Raman spectra were measured using a high resolution Raman spectrometer (LabRAM HR Evolution, Horiba JY, Kyoto, Japan) and a continuous wave laser with a wavelength of 514.5 nm and an excitation power of 5 mW. Using an SPS furnace, the green bodies were sintered in the mold for 15 min at 688 K and 60 MPa under uniaxial pressure (SPS-20 T-10IV, Shanghai Chen Hua Technology Co., Ltd., Shanghai, China). Using a Thermoelectric Test System (LINSEIS LSR-3, Selb, Germany), the Seebeck coefficient (*S*) and electrical conductivity (σ) were measured simultaneously in the temperature range of 300 to 550 K with a 50 K step.

## 3. Results and Discussion

### Structure and Morphology of Bi${}_{2}$Te${}_{3}$ Nanosheets

**Figure 2:**
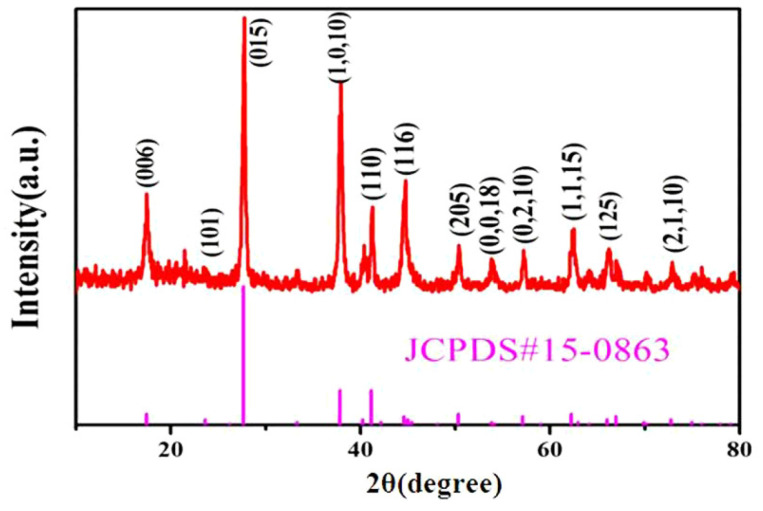
XRD pattern of the as-prepared Bi${}_{2}$Te${}_{3}$ nanostructures.

Figure 2 shows the XRD (Bruker D8 Advance) patterns of the produced Bi${}_{2}$Te${}_{3}$ nanostructures. The Bi${}_{2}$Te${}_{3}$ rhombic lattice is represented by all diffraction peaks in the XRD data (JCPDS: 15-0863). The stylized peaks are readily correlated with the planes of the Bi${}_{2}$Te${}_{3}$ rhombic lattice phases (015), (1,0,10), (110), (205), (0,2,10), and (1,1,15) [12,19,20,21,22,26,50,59,60,61,62,63,64].

**Figure 3:**
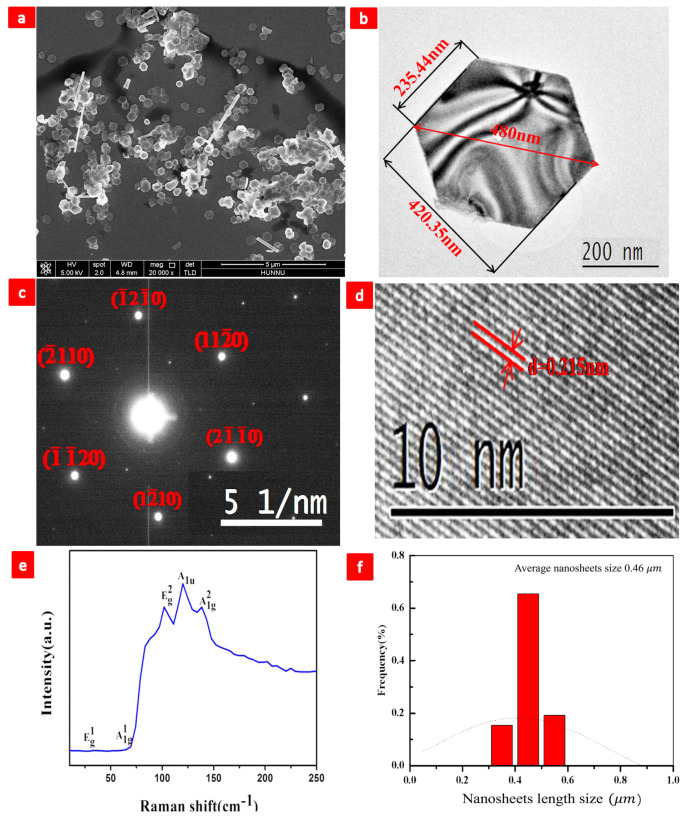
(**a**) SEM of Bi${}_{2}$Te${}_{3}$ nanosheets, (**b**) TEM of Bi${}_{2}$Te${}_{3}$ nanosheets, (**c**) SAED of Bi${}_{2}$Te${}_{3}$ nanosheets, (**d**) HRTEM of Bi${}_{2}$Te${}_{3}$ nanosheets, (**e**) Raman of Bi${}_{2}$Te${}_{3}$ nanosheets, and (**f**) average nanosheet size, 0.46 µm.

Figure 3a depicts a large-scale SEM (FEI, NovaSEM-450) nanosheet of Bi${}_{2}$Te${}_{3}$ with a uniform hexagonal shape. Figure 3b depicts a transmission electron microscopy (TEM, FEI Tecnai F20) image of a single Bi${}_{2}$Te${}_{3}$ nanosheet with a typical flat surface and sharp edges. The 230–420 nm size of these Bi${}_{2}$Te${}_{3}$ nanosheets is sufficient for Raman investigation utilizing an optical microscope. Figure 3c depicts the selected area electron diffraction pattern, which reveals a hexagonally symmetric diffraction dot pattern, demonstrating that Bi${}_{2}$Te${}_{3}$ nanosheets are single-crystal. Figure 3d is a HRTEM (FEI Tecnai F20) image with great resolution [12,19,20,21,22,26,50,59,60,61,62,63]. The flat spacing recorded is 0.215 nm. Single-crystal nanosheets developed along the axis projection of the [0001] zone of the Bi${}_{2}$Te${}_{3}$ hexagonal reciprocal lattice are shown in Figure 3b,c [12,19,20,21,22,26,50,59,60,61,62,63]. Figure 3e depicts the Raman spectrum (LabRAM HR Evolution, Horiba JY) of a single Bi${}_{2}$Te${}_{3}$ nanosheet at room temperature. The peak positions correspond to those previously recorded for crystalline Bi${}_{2}$Te${}_{3}$ in bulk [12,19,20,21,22,26,50,59,60,61,62,63]. Figure 3f shows the average size of nanosheets, which is 0.46 µm.

## 4. Morphological Evolution of Bi${}_{2}$Te${}_{3}$ Nanosheets

In investigating the influence of various factors on the morphology of Bi${}_{2}$Te${}_{3}$ nanosheets during synthesis, a series of comparative experiments was performed.

### 4.1. Influences of the Reaction Temperature

**Figure 4:**
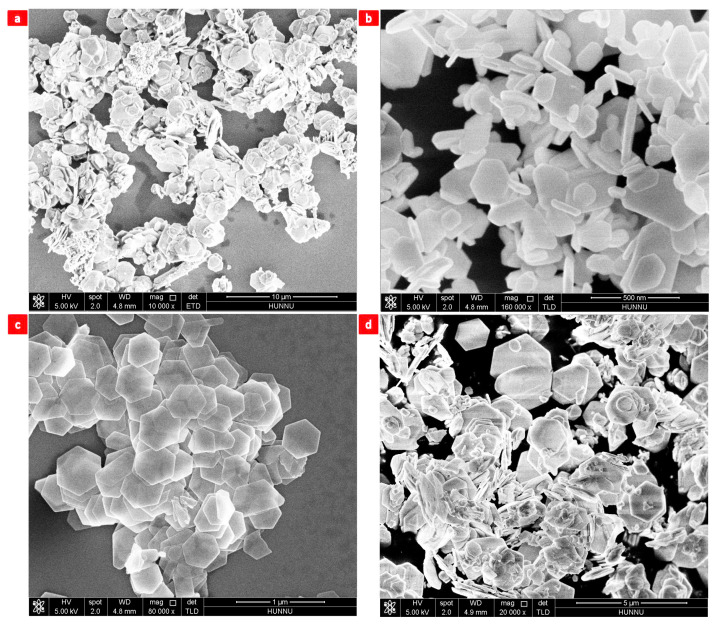
Reaction temperatures: (**a**) 140 °C, (**b**) 160 °C, (**c**) 180 °C, and (**d**) 200 °C.

A temperature range of 140 °C to 200 °C was used to investigate the effects of temperature on the morphological evolution of Bi${}_{2}$Te${}_{3}$ nanosheets [19,20,21,26,61]. The morphologies of the reaction products are shown in Figure 4a–d.

The reaction temperature was set at 140 °C. One morphology (nanoparticles) of the products is presented in Figure 4a. When the reaction temperature is 160 °C, the reaction products show two main morphologies (Figure 4b), namely, nanowires and hexagonal nanosheets. At 180 °C, the reaction products were single-crystal hexagonal Bi${}_{2}$Te${}_{3}$ nanosheets with a regular morphology and uniform size (Figure 4c). At 200 °C, the reaction product is a nanocluster (Figure 4d). Table 1 shows the effects of reaction temperature.

As shown in Table 1, the product’s form changes from nanoparticles to nanosheets as the temperature rises. Based on the Gibbs–Thomson effect, smaller crystallites contain greater Gibbs free energy. Consequently, the chemical process for creating Bi${}_{2}$Te${}_{3}$ nanosheets takes more energy, and lower temperatures cannot provide enough energy to turn reactants into Bi${}_{2}$Te${}_{3}$ nanosheets [19,20,21,26,61].

Optimized analysis of the morphology of Bi${}_{2}$Te${}_{3}$ nanosheets by this group of experiments of reaction temperature shows that the morphology of the products grows gradually from nanoparticles to nanosheets with the increase in temperature (Table 1). Given the Gibbs–Thomson effect, the smaller the grain, the larger the Gibbs free energy. In addition, when the Gibbs free energy is larger, the grain becomes increasingly unstable. When the temperature continues to increase, the Gibbs free energy of the nanoparticles also continues to increase. In ensuring the stability of the system, the nanoparticle can only increase its own diameter to reduce the Gibbs free energy, thereby making the system stable [19,20,21,26,59,60,61,62,63,64].

The chemical reaction process of chemical synthesis includes both ionic and atomic mechanisms. During ionic reactions, atoms are first reduced to unity by a strong reducing agent and then combined to form. During an atomic reaction, it is reduced to atoms by a strong reducing agent and then produced in a direct atomic reaction with the metal [65]. When the reaction temperature is low and the reaction time is short, the ionic reaction dominates, while when the reaction temperature rises and the reaction time increases, the atomic reaction dominates [66]. This reaction process is energy intensive, and lower temperatures are clearly not sufficient to provide enough energy to allow the product to fully react to Bi${}_{2}$Te${}_{3}$ [67].

### 4.2. Influences of the Molar Mass of NaOH

The alkaline surroundings affect the final morphology of the nanomaterials. A series of experiments was conducted to investigate the effect of NaOH solubility on the final morphology of nanomaterials [19,20,21,22,26,59,60,61,62,63].

**Figure 5:**
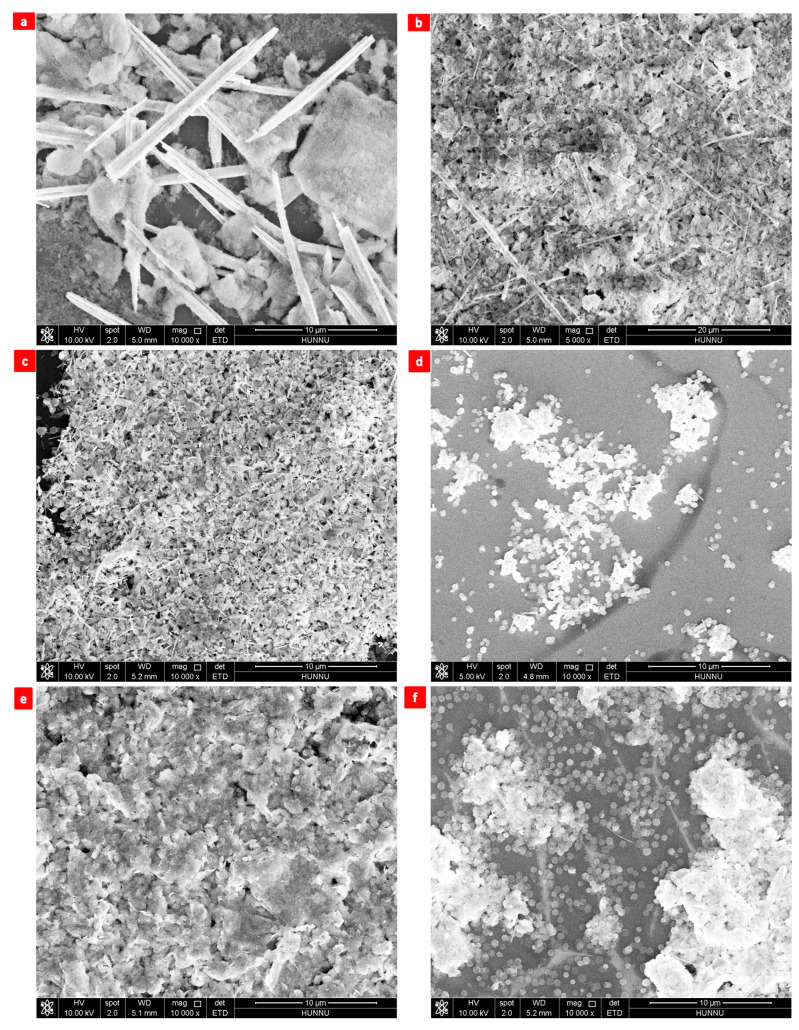
Molar mass of NaOH: (**a**) 0.0 mmol, (**b**) 5.0 mmol, (**c**) 10.0 mmol, (**d**) 20.0 mmol, (**e**) 40.0 mmol, and (**f**) 17.8 mmol KOH.

When the reaction was carried out without NaOH, no nanosheets were obtained, and the products were mainly strips of nanorods and nanoclusters (Figure 5a). At 5.0 mmol NaOH, nanosheets were observed in the product (Figure 5b), along with many residual rod-like nanorods [26,59,60,61,62,63]. When the mass of NaOH increases to 10.0 mmol, the product contains nanorods (Figure 5c) [19,20,21,22,26,59,60,61,62,63]. At 20.0 mmol NaOH, the product was Bi${}_{2}$Te${}_{3}$ nanosheets with a uniform size, well-defined profile, and sharp edges (Figure 5d) [26,59,60,61,62,63]. When NaOH was 40.0 mmol, the product was nanoclusters (Figure 5e). Under 17.8 mmol NaOH substituted for KOH, the product was Bi${}_{2}$Te${}_{3}$ nanoclusters (Figure 5f) [59,60,61,62,63]. Based on these experimental results, 20.0 mmol of NaOH was suitable for the preparation of single-crystal hexagonal Bi${}_{2}$Te${}_{3}$ nanosheets with a regular morphology and uniform size. Analysis of the impact of NaOH on the morphology of Bi${}_{2}$Te${}_{3}$ nanosheets is presented in Table 2.

In decreasing the acidolysis of the process, an alkaline material is required. As shown in Figure 5a–f, sodium hydroxide would increase the alkaline concentration of the process, resulting in hexagonal Bi${}_{2}$Te${}_{3}$ nanosheets with uniform dimensions, well-defined profiles, and sharp edges. Therefore, OH${}^{-}$ can promote cation release in the solution and steer the development of non-equilibrium crystals with high monomer concentration toward certain crystal planes.

The pH of the solution strongly influences the growth of the crystalline nanostructures: (1) The evolution of different nanostructures, starting from Bi${}_{2}$Te${}_{3}$ primary particles, can be explained by considering two different mechanisms viz. Ostwald ripening (OR) and orient attachment (OA) mechanisms. The OR mechanism is a thermodynamically driven spontaneous process in which energetic factors will cause the larger particles to grow at the expense of smaller grains. Here, smaller primary particles dissolves and re-precipitates on the surface of larger particles, yielding surfaces will be eliminated, leading to lowering the surface free energy, obeying thermodynamic rules [68]. A perfect OA process will yield defect-free and single crystalline nanoparticles through the attachment of primary particles in an irreversible and strongly oriented manner. An imperfect OA mechanism yields nanoparticles with surface defects, which acts as a speckle for secondary nucleation [69]. The SEM image of the as-synthesized nanoclusters and nanorods in Figure 5a,b shows several surface defects, revealing that the imperfect orient attachment mechanism leads to the formation of these hierarchical structures; (2) under alkaline conditions, the higher concentration of OH${}^{-}$ ions leads to well-solvated ions, resulting in an enhanced rate of formation of Bi${}_{2}$Te${}_{3}$. A detailed study was carried out to find the reaction mechanism of formation of these nanorod architectures and it is found that Bi${}_{2}$Te${}_{3}$ nanorods are evolved out of Bi${}_{2}$Te${}_{3}$ nanowires. Initially, Bi${}^{3+}$ and Te${}^{2-}$ ions combine to form Bi${}_{2}$Te${}_{3}$ crystals and these tiny crystals grow into Bi${}_{2}$Te${}_{3}$ nanoplates in the subsequent stage; (3) under highly alkaline conditions, Bi${}_{2}$Te${}_{3}$ nanowires along with nanoplates are formed with the length of the nanosheets ranging [70,71]. Here also, the observed nanoclusters can be explained by considering the intrinsic crystal structure properties.

### 4.3. Influences of the Surfactants

The surfactants can effectively control the morphological evolution of Bi${}_{2}$Te${}_{3}$ nanosheets. By optimizing the concentration of surfactants, such as CTAB, EDTA, and SDBS, these as-synthesized products exhibit exclusively different morphologies (nanowire, nanoparticles, and nanosheets) with unchanged conditions [26,59,60,61,62,63].

**Figure 6:**
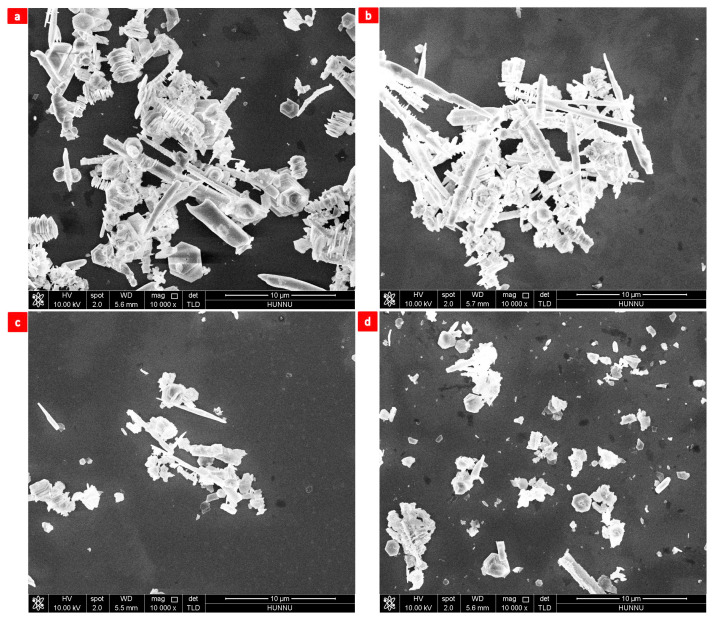
Surfactants: (**a**) no surfactant, (**b**) CTAB, (**c**) EDTA, and (**d**) SDBS.

When no surfactant is used, the products are nanoclusters and nanorods (Figure 6a). When the surfactant is CTAB, the obtained product is composed of nanorods with burrs growing around the nanorods (Figure 6b). By contrast, when the surfactant was EDTA, the product was nanorods (Figure 6c). Based on the abovementioned finding, the obtained products are composed of nanosheets and nanorods. Many residual rods are identified when the surfactant is SDBS (Figure 6d).

The preceding research indicates that surfactants influence the form and substance of the product. Surfactants, such as CTAB and EDTA, can boost the amount of nanorods in a product. These findings could be due to the detergents binding and covering the initial nanocluster production.

Surfactants adsorb and cover the first produced nanoclusters, thereby lowering the Te and Bi combination. In producing hexagonal Bi${}_{2}$Te${}_{3}$ nanosheets with a constant form and size, the surfactant might be PVP in a 1.0 g proportion. The impact of surfactants on the synthesis of Bi${}_{2}$Te${}_{3}$ is presented in Table 3.

### 4.4. Influences of the Reaction Time

**Figure 7:**
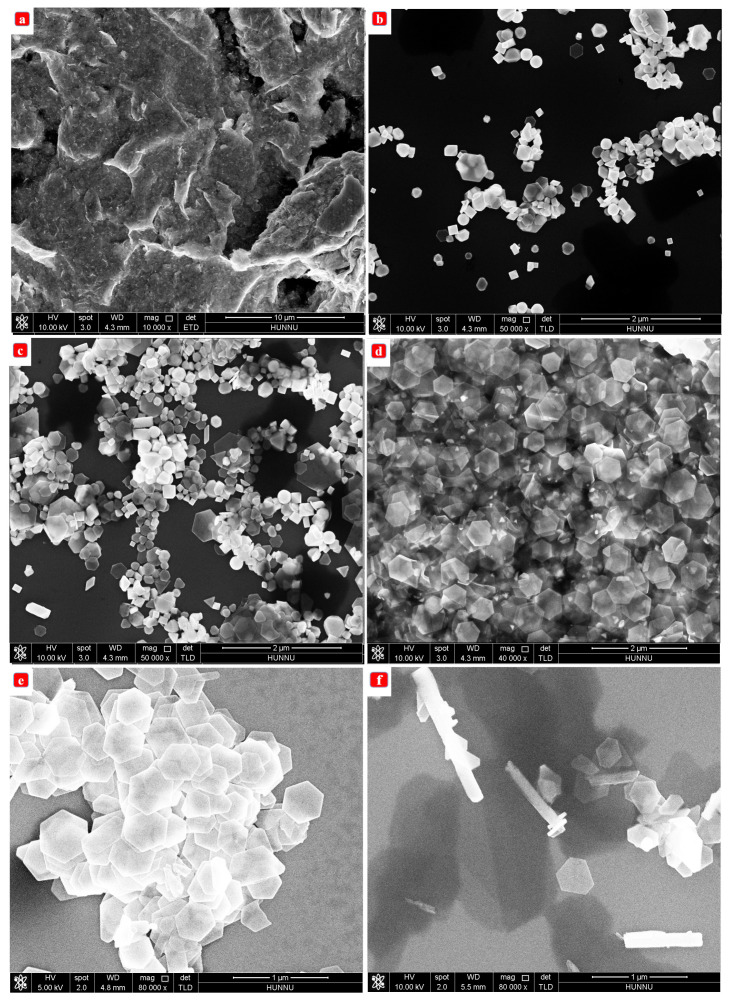
Reaction time: (**a**) 3 h, (**b**) 6 h, (**c**) 12 h, (**d**) 24 h, (**e**) 36 h, and (**f**) 48 h.

Considering that reaction time was an important aspect in the development process, we investigated the growth of Bi${}_{2}$Te${}_{3}$ nanosheets using SEM images and created a time-dependent experiment of the single variable while holding the other factors constant [19,20,21,22,26,50,59,60,61,62,63,64]. Figure 7a–f present typical SEM images of the products synthesized at 3, 6, 12, 24, 36, and 48 h, respectively.

When the reaction time was 3 h, nanoparticles appeared (Figure 7a), and when the reaction time was 6 h, a small number of nanoparticles and nanosheets appeared (Figure 7b). At 12 h, Bi${}_{2}$Te${}_{3}$ nanoparticles were observed (Figure 7c); at 24 h, the products and nanoparticles of Bi${}_{2}$Te${}_{3}$ nanosheets were poor (Figure 7d); at 36 h, hexagonal Bi${}_{2}$Te${}_{3}$ nanosheets with a uniform size and sharp edges were prepared (Figure 7e) [59,60,61,62,63]. When the reaction time was 48 h, nanorods were observed (Figure 7f) [26,50,59,60,61,62,63]. An analysis of the impact of the reaction time on the morphology of Bi${}_{2}$Te${}_{3}$ nanosheets is presented in Table 4.

As shown in Table 4, the number of nanosheets increased when nanoparticles gradually disappeared. With a longer reaction time, the diffraction peaks of the samples become sharper. Therefore, the crystallinity of nanosheets increases with the increase in reaction time. Based on these experimental results, the optimal time for the preparation of single-crystal hexagonal Bi${}_{2}$Te${}_{3}$ nanosheets with a regular morphology and uniform size is 36 h.

Under relatively mild conditions, the intrinsic growth morphology of Bi${}_{2}$Te${}_{3}$ nanosheets exhibits a layered shape because of the lattice structure. At the outset of the process, freshly generated Te nuclei absorbed Bi${}_{2}$Te${}_{3}$ grains and formed a hexagonal prototype. Using the surfactant PVP, the hydrophobic end is adsorbed on the (001) surface, restricting the growth of Bi${}_{2}$Te${}_{3}$ on the (001) surface. Consequently, the development rate of Bi${}_{2}$Te${}_{3}$ nanosheets along the a- or b-axes is quicker than that along the c-axis, leading to the synthesis of hexagonal nanosheets [26,50,59,60,61,62,63].

## 5. Transport Properties of Bi_2_Te_3_ Solid-State Samples

An appropriate synthesis formula was identified by studying the factors affecting the synthesis and formed hexagonal Bi${}_{2}$Te${}_{3}$ nanosheets with a mean particle size distribution of 0.46 µm.

### 5.1. Measurement of Solid-State Sample Density

**Figure 8:**
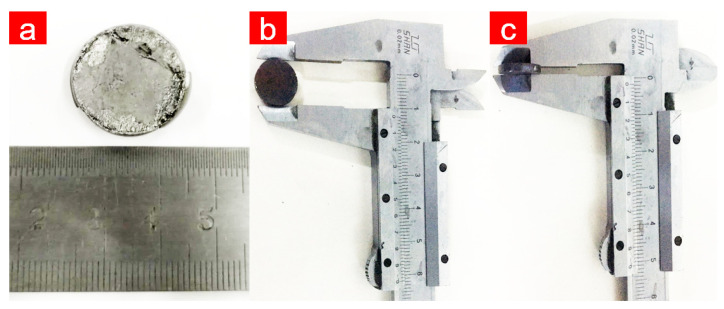
(**a**) Bi${}_{2}$Te${}_{3}$ solid-state samples, (**b**) solid-state sample diameter, and (**c**) solid-state sample thickness.

Following controlled synthesis analysis, Bi${}_{2}$Te${}_{3}$ nanopowders were crushed into solid specimens by spark plasma sintering (SPS-20 T-10IV, Shanghai Chen Hua Technology Co., Ltd.) and using a thermoelectric test system (LINSEIS LSR-3). Figure 8a shows Bi${}_{2}$Te${}_{3}$ solid-state samples with a diameter of 20.00 mm and a height of 5.00 mm. The wedge specimens were ground to a regular size of 12.50 mm in diameter and 1.95 mm in thickness (Figure 8b,c).

The weight *M*${}_{0}$, diameter *D*, and thickness *H* of the block was tested three times using a scale and rotary encoder counters, and the average data were determined. As shown in Table 5, the collected data were fed into the density equation to compute the volume, density, and relative density of the circular block.

The density of circular Bi${}_{2}$Te${}_{3}$ solid-state samples was 7.2456 g/cm${}^{3}$, whereas that of the pure Bi${}_{2}$Te${}_{3}$ block was 7.7 g/cm${}^{3}$, with a 93.9% relative density of the specimen block. This result indicates that the Bi${}_{2}$Te${}_{3}$ solid-state samples are dense, which is advantageous for boosting the electrical conductivity.

### 5.2. Electrical Conductivity (σ) of the Bi${}_{2}$Te${}_{3}$ Solid-State Samples

**Figure 9:**
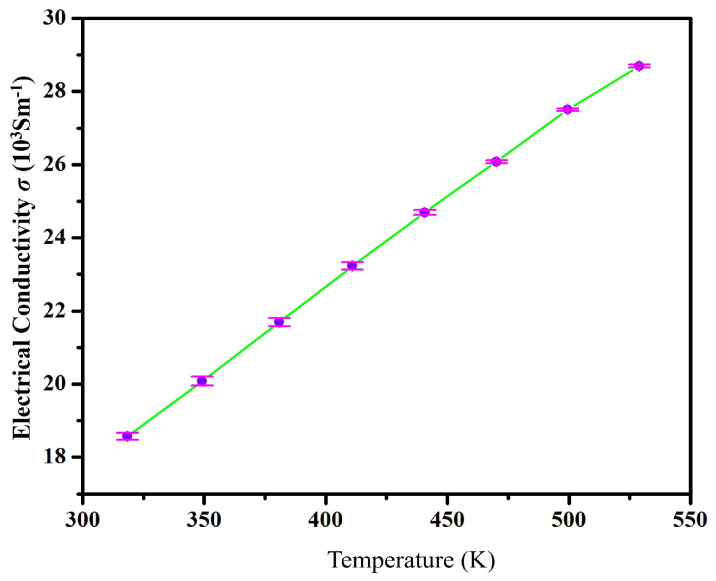
Characteristic of electrical conductivity σ with regard to temperature dependency.

The temperature of hot pressed Bi${}_{2}$Te${}_{3}$ bulk specimens was plotted against their conductivity. As shown in Figure 9, the conductivity of a Bi${}_{2}$Te${}_{3}$ solid-state sample increases with the increase in temperature from room temperature to 550 K. The conductivity continues σ to increase with temperature until it reaches its maximum value of 28.69 × 10${}^{3}$ Sm${}^{-1}$. Based on a previous study, the conductivity of Bi${}_{2}$Te${}_{3}$ solid-state samples increases with temperature. At low and moderate temperatures, the carrier increases exponentially because of the impurity excitation caused by semiconductor materials, but conductivity increases exponentially as temperatures rise.

### 5.3. Seebeck Coefficient (*S*) of the Bi${}_{2}$Te${}_{3}$ Solid-State Samples

**Figure 10:**
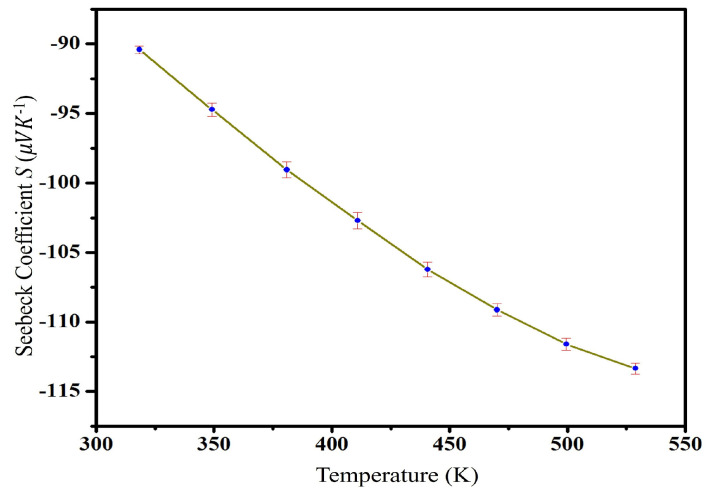
Difference in the temperature dependency of the Seebeck coefficient (*S*).

The Bi${}_{2}$Te${}_{3}$ solid-state samples are made of an N-type semiconductor material because the Seebeck coefficients (Figure 10) are negative. Conductivity decreased with the increase in temperature until it reaches its lowest value of −113.3 µVK${}^{-1}$. The sample’s Seebeck coefficient *S* drops as the temperature rises because of the nanoscale level, and the crystal size of the powdered sample is tiny, which causes many defects and increases carrier scattering, thereby increasing the scattering factor and Seebeck coefficient.

The optimal Bi${}_{2}$Te${}_{3}$ nanosheets obtained at the growth conditions including reaction temperature, surfactant PVP dosage, the molar mass of NaOH, and the reaction time are 120 °C, 1.0 g, 20.0 mmol, and 36 h, respectively. The novelty of this paper is the green synthesis and evolution of morphology: (1) Without using strong acids (e.g., HNO${}_{3}$) [72], strong bases (e.g., KOH) [73], or strong reducing agents (e.g., N${}_{2}$H${}_{4}$) [74], the nanosheets with regular hexagonal morphology and uniform size were generated by the weak reducing effect of EG and low synthesis temperature. (2) Evolution of morphology: the effects of reaction time, reaction temperature, alkaline environment and surface activity on the morphology of the reaction products are discussed in detail [75]. The precise control of growth conditions allows for accurately adjusting the nucleation and diffusion rates of Bi and Te and the growth rate of the Bi${}_{2}$Te${}_{3}$ nanosheets [76,77].

## 6. Conclusions

In conclusion, we have successfully synthesized Bi${}_{2}$Te${}_{3}$ nanosheets via a hydro-thermal method. The average length of these nanoplates is 0.46 µm. The reaction temperature, the molar mass of NaOH, and surfactant PVP dosage play important roles on the growth of Bi${}_{2}$Te${}_{3}$ nanocrystals. PVP and NaOH are a first and necessary one for synthetics of Bi${}_{2}$Te${}_{3}$ nanosheets. The thickness of Bi${}_{2}$Te${}_{3}$ nanosheets via adjusting the weight of NaOH. The reaction time 36 h is a little long, but it precisely controls the growth of Bi${}_{2}$Te${}_{3}$ nanosheets. No improvement in transport properties compared with those of Refs. [66,75,76,77,78,79]. The self-repairing capability of Bi${}_{2}$Te${}_{3}$ nanosheets not only provides insight into the growth of Bi${}_{2}$Te${}_{3}$ nanosheets, but also enhances and completes the method for synthesizing Bi${}_{2}$Te${}_{3}$ nanosheets.

## Figures and Tables

**Table 1:** The morphology of Bi${}_{2}$Te${}_{3}$ was altered by a range of reaction temperatures.

	TeO${}_{2}$	BiCl${}_{3}$	NaOH	PVP (K-30)	t(h)	T(°C)	Morphology
(a)	3.0 mmol	2.0 mmol	20.0 mmol	1.0 g	36 h	140 °C	nanoparticles
(b)	3.0 mmol	2.0 mmol	20.0 mmol	1.0 g	36 h	160 °C	nanoparticles and nanosheets
(c)	3.0 mmol	2.0 mmol	20.0 mmol	1.0 g	36 h	180 °C	nanosheets
(d)	3.0 mmol	2.0 mmol	20.0 mmol	1.0 g	36 h	200 °C	nanoclusters

**Table 2:** Influence of NaOH on the morphology for Bi${}_{2}$Te${}_{3}$.

	TeO${}_{2}$	BiCl${}_{3}$	PVP	T(°C)	t(h)	NaOH	Morphology
(a)	3.0 mmol	2.0 mmol	1.0 g	180 °C	36 h	0.0 mmol	nanoclusters and nanorods
(b)	3.0 mmol	2.0 mmol	1.0 g	180 °C	36 h	5.0 mmol	nanorods
(c)	3.0 mmol	2.0 mmol	1.0 g	180 °C	36 h	10.0 mmol	nanowires and nanosheets
(d)	3.0 mmol	2.0 mmol	1.0 g	180 °C	36 h	20.0 mmol	nanosheets
(e)	3.0 mmol	2.0 mmol	1.0 g	180 °C	36 h	40.0 mmol	nanoclusters
(f)	3.0 mmol	2.0 mmol	1.0 g	180 °C	36 h	KOH	nanoclusters

**Table 3:** Effect of surfactants on the morphology for Bi${}_{2}$Te${}_{3}$.

	TeO${}_{2}$	BiCl${}_{3}$	T(°C)	t(h)	NaOH	Surfactants	Morphology
(a)	3.0 mmol	2.0 mmol	180 °C	36 h	20.0 mmol	0.0 g	nanoclusters and nanorods
(b)	3.0 mmol	2.0 mmol	180 °C	36 h	20.0 mmol	CTAB	nanorods
(c)	3.0 mmol	2.0 mmol	180 °C	36 h	20.0 mmol	EDTA	nanorods
(d)	3.0 mmol	2.0 mmol	180 °C	36 h	20.0 mmol	SDBS	nanosheets and nanorods

**Table 4:** The morphology of Bi${}_{2}$Te${}_{3}$ was modified by a succession of reaction times.

	TeO${}_{2}$	BiCl${}_{3}$	NaOH	PVP	T(°C)	t(h)	Morphology
(a)	3.0 mmol	2.0 mmol	20.0 mmol	1.0 g	180 °C	3 h	nanoparticles
(b)	3.0 mmol	2.0 mmol	20.0 mmol	1.0 g	180 °C	6 h	nanoparticles and nanosheets
(c)	3.0 mmol	2.0 mmol	20.0 mmol	1.0 g	180 °C	12 h	nanoparticles and nanosheets
(d)	3.0 mmol	2.0 mmol	20.0 mmol	1.0 g	180 °C	24 h	nanosheets
(e)	3.0 mmol	2.0 mmol	20.0 mmol	1.0 g	180 °C	36 h	nanosheets
(f)	3.0 mmol	2.0 mmol	20.0 mmol	1.0 g	180 °C	48 h	nanorods

**Table 5:** Density of the circular Bi${}_{2}$Te${}_{3}$ solid-state samples.

Parameters Name	*M*${}_{0}$(g)	*D*(mm)	*H*(mm)	*V*(cm${}^{3}$)	ρ(g/cm${}^{3}$)	Relative Density
Measured value	1.733	12.50	1.95	0.23918	7.2456	93.9%

## Data Availability

Not applicable.
